# Translation, but not transfection limits clinically relevant, exogenous mRNA based induction of alpha-4 integrin expression on human mesenchymal stem cells

**DOI:** 10.1038/s41598-017-01304-3

**Published:** 2017-04-24

**Authors:** Adam Nowakowski, Anna Andrzejewska, Johannes Boltze, Franziska Nitzsche, Li-li Cui, Jukka Jolkkonen, Piotr Walczak, Barbara Lukomska, Miroslaw Janowski

**Affiliations:** 1 NeuroRepair Department, Mossakowski Medical Research Centre, Polish Academy of Sciences, Warsaw, Poland; 2 Fraunhofer-Institute for Cell Therapy and Immunology, Department of Cell Therapy, University of Leipzig, Leipzig, Germany; 3 Translational Centre for Regenerative Medicine, University of Leipzig, Leipzig, Germany; 4 Fraunhofer Research Institution of Marine Biotechnology, Department of Translational Medicine and Cell Technology, Lübeck, Germany; 5 Institute for Medical and Marine Biotechnology, University of Lübeck, Lübeck, Germany; 6 Stroke Recovery Laboratory, Institute of Clinical Medicine - Neurology, University of Eastern Finland, Kuopio, Finland; 7 Neurocenter, Neurology, University Hospital of Kuopio, Kuopio, Finland; 8 Russell H. Morgan Dept. of Radiology and Radiological Science, Division of MR Research, The Johns Hopkins University School of Medicine, Baltimore, USA; 9 Institute for Cell Engineering, The Johns Hopkins University School of Medicine, Baltimore, USA; 10 Department of Neurology and Neurosurgery, Faculty of Medical Sciences, University of Warmia and Mazury, Olsztyn, Poland

## Abstract

Mesenchymal stem cells (MSCs) represent promising resource of cells for regenerative medicine in neurological disorders. However, efficient and minimally invasive methods of MSCs delivery to the brain still have to be developed. Intra-arterial route is very promising, but MSCs are missing machinery for diapedesis through blood-brain barrier. Thus, here we have tested a mRNA-based method to induce transient expression of ITGA4, an adhesion molecule actively involved in cell extravasation. We observed that transfection with an ITGA4-mRNA construct bearing a conventional cap analogue (7-methylguanosine) failed to produce ITGA4 protein, but exogenous ITGA4-mRNA was detected in transfected MSCs. This indicates that not transfection, but rather translation being the major roadblock. Stabilization of ITGA4-mRNA with SSB proteins resulted in ITGA4 protein synthesis in HEK293 cells only, whereas in MSCs, satisfactory results were obtained only after using an anti-reverse-cap-analogue (ARCA). The presence of ITGA4 protein in MSCs was transient and lasted for up to 24 h after transfection. Membranous location was confirmed by flow cytometry of viable non-permeabilized cells using anti-ITGA4 antibody. The mRNA-based expression of *itga4* transgene is potentially sufficient for diapedesis after intra-arterial delivery. To conclude, mRNA-based engineering of stem cells is a rapid and integration-free method and attractive from the perspective of potential future clinical application.

## Introduction

There is a growing demand for regenerative medicine solutions allowing repair or even replacement of strained or injured tissues, particularly as societies are ageing. Progress in this field including cell therapy and tissue engineering is remarkable, but neurological diseases pose a special problem for regenerative medicine. Unlike for most other organs, the unique role and function of the central nervous system (CNS) makes organ transplantation unfeasible. Moreover, tissue replacement strategies are hampered by the CNS complexity^1^ while the previous failure of drug-based neuroprotection adds to the grim prognosis^2^. Due to its high frequency and severe sequel such as long-term disability, stroke results in an enormous social and financial burden to societies. Cell therapies are among the most promising options for stroke which can be applied beyond the extremely narrow therapeutic time window offered by thrombolysis. Hence, translation of experimental cell transplantation approaches into clinically applicable therapies is a currently ongoing process^3^. The relative abundance, safety as well as easy access to autogenic sources make mesenchymal stem cells (MSCs) a very good candidates for use in regenerative strategies^4^. There are many reports indicating that the application of exogenous MSCs brings beneficial therapeutic effects in neurological disorders^5^ and other ailments such as diabetes type I^6^, haematological^7^, liver^8^, and cardiac diseases^9^, validated by clinical trials reporting preliminary evidence for favourable outcomes^10, 11^. The beneficial results are thought to be due to trophic and immunomodulatory effects exerted by the plethora of biologically active compounds produced by MSCs^12^.

There are several potential routes to target MSCs to the ischemic brain regions including intracerebral^13^, intraventricular^14^, intravenous^15^ and intraarterial^16–18^. The first two routes require craniotomy and direct puncture of brain parenchyma. On the other hand, the intravenous route is highly unspecific as it distributes cells throughout the circulation, thus requiring large doses of cells, as well as risk of side effects related to target accumulation of injected cells with pulmonary embolism being a prominent example^19^. Nevertheless, systemic delivery of therapeutic MSCs seems to be a minimally invasive not only for neurological purposes (especially an intra-arterial route) but also for relatively “hard-to-reach” organs such as the pancreas i.e. in diabetes type I^20^ and pancreatic cancer^21^. Its widespread applicability is anticipated once the some obstacles constituted by the inefficient vascular extravasation of naïve MSCs in the target region is solved. First, insufficient extravasation limits the number of MSCs available at the lesion site. Second, size of these cells exceeds that of capillaries and their intra-arterial injection introduces a risk of micro-occlusions and ischemia by entrapment in the vessel lumen^22, 23^. This may severely compromise the consistent therapeutic benefits exerted by MSCs as shown in numerous animal models of stroke^24^.

Hence, diapedesis fostering fast clearance through transendothelial extravasation is of utmost importance. Furthermore, DNA-based genetic engineering of glial restricted precursors (GRPs) toward the expression of VLA-4, physiologically involved in leukocyte extravasation^25^ was sufficient to dock GRPs to the vessel wall^26^. The improvement of migratory properties of MSCs including extravasation can be effectively accomplished by genetic engineering such as overexpression of epidermal growth factor receptor using viral vector^27^. However, viral vectors are linked to significant safety concerns and despite high transfection efficiency, they are unlikely to find wide clinical use^28^. Additionally, long-lasting transgene expression extending beyond the time required for crossing vascular walls is undesirable. For this purpose, safer, transient and thereby clinically more acceptable methods for MSC engineering are highly desired. Several examples of pDNA-based MSC modifications exist^29–32^. However, these methods do not yield high transfection efficiency.

Naïve MSCs were shown to express integrin β_1_ subunit (ITGB-1) but do not express the integrin α_4_ (ITGA4) subunit which is required to produce the complete VLA-4 heterodimer^33^. Previously, it was shown that *itga4* gene overexpression in MSCs using viral vector led to proper routing of the protein, physiological interactions with the endogenous ITGB-1 subunit and assembling of functional VLA-4 heterodimers^34^. Thus, we decided to follow this strategy and engineer MSCs to overexpress the *itga4* gene. Since viral-based methods have unfavourable safety profiles and DNA transfection is inefficient, we decided to utilize mRNA which was shown as an effective tool for genetic cell engineering^35, 36^. Since mRNA transfection is an integration-free method, inducing short-lasting expression and high transfection efficiency, it is ideal for the targeted transient induction of *itga4* gene expression in MSCs. The overview of the article is presented in Supplementary Figure 1.

## Results

### DNA Plasmid transfection

**Figure 1:**
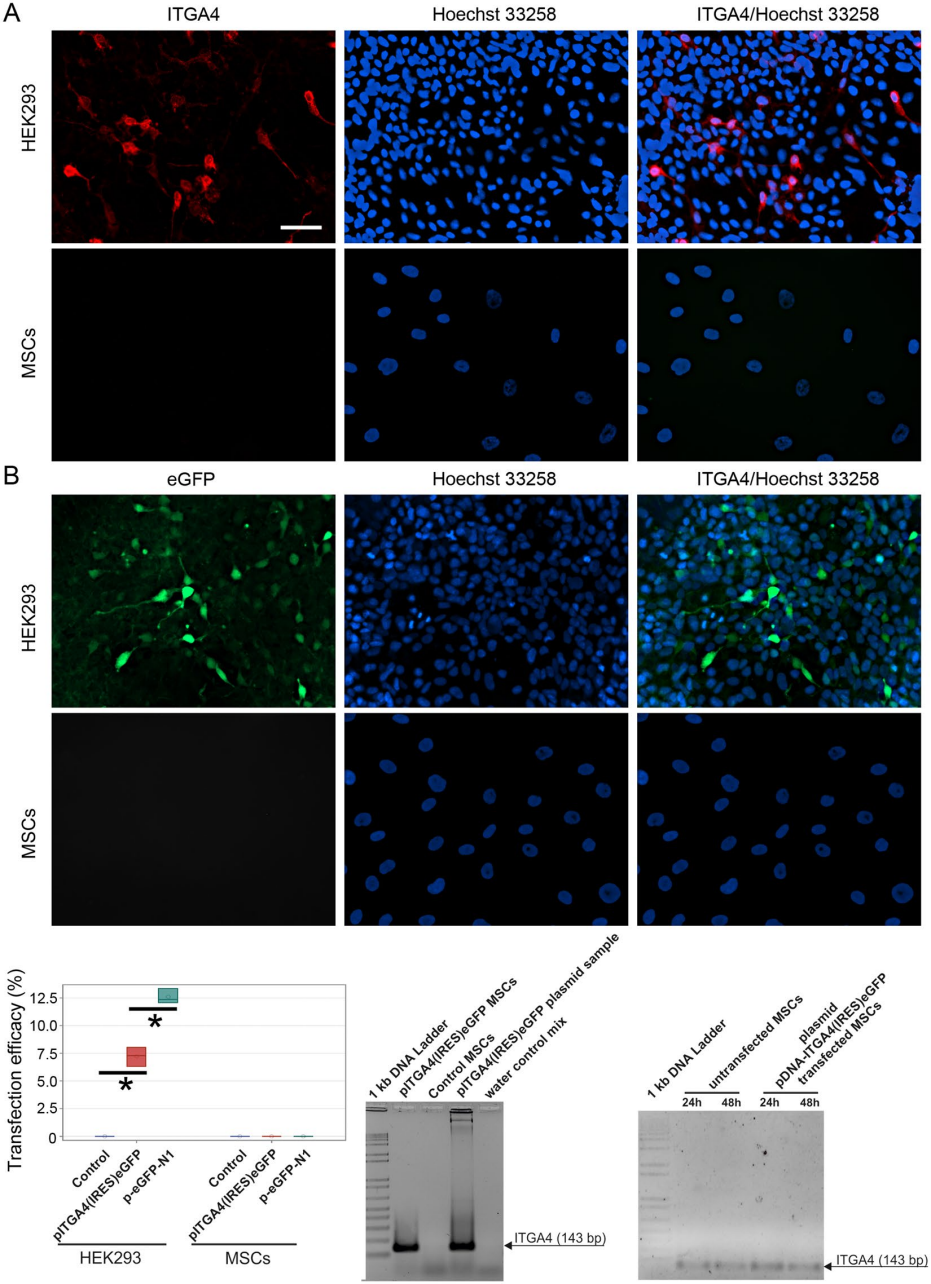
Plasmid DNA-based transfections of MSCs and HEK293 cells. MSCs and HEK293 visualized 24 h post transfection by immunofluorescence after pITGA4(IRES)eGFP plasmid (**A**) and p-eGFP-N1 plasmid (**B**). Quantification of percentage of cells expressing transgenes [pITGA4(IRES)eGFP/red/, p-eGFP-N1/green/] (**C**). PCR for presence of pITGA4(IRES)eGFP plasmid in MSCs 24 h after transfection (**D**). PCR for presence of *itga4* mRNA in MSCs transfected with pITGA4(IRES)eGFP (**E**). The arrows point on PCR-product corresponding *itga4* gene (143 bp). Error bars are box-and-whiskers plots containing the mean, quartiles (box) and minimum and maximum observations (whiskers). Transfection efficiencies have been calculated from three independent experiments. Scale bar located in the top left image: 20 µm.

Transfection with pITGA4(IRES)eGFP plasmid resulted in no detection of ITGA4 protein in MSCs (0%), while ITGA4 protein was present in 7.2% of HEK293 cells (p < 0.05) (Fig. 1A). The production of ITGA4 protein by HEK293 cells allowed us to confirm the proper vector design so the produced protein is detectable by immunohistochemistry. In order to clarify whether the failure of pITGA4(IRES)eGFP plasmid expression in MSCs was due to the large construct size, we performed a transfection with smaller p-eGFP-N1 plasmid coding solely eGFP protein, and we also did not find any presence of eGFP protein in MSCs (0%), while the eGFP protein was present in 12.6% of HEK293 cells (Fig. 1B). Expectedly, we found the higher expression of smaller p-eGFP-N1 plasmid than larger pITGA4(IRES)eGFP plasmid in HEK293 cells (p < 0.05), while no expression of both plasmids was found in MSCs (Fig. 1C).

To investigate the reason for the lack of plasmid DNA expression in MSCs, we assessed whether the pITGA4(IRES)eGFP plasmid was effectively internalized by the MSCs. Cell lysates were tested by PCR with primers detecting pITGA4(IRES)eGFP plasmid DNA 24 h after transfection. Its presence in the cells was confirmed (Fig. 1D). Nevertheless, detection of ITGA4-mRNA revealed only basal endogenous ITGA4-mRNA with indistinguishable differences in mRNA levels in transfected and naïve MSCs at 24 and 48 h after transfection (Fig. 1E). This indicates that not transfection itself, but transcription is the major roadblock to achieve efficient protein production in DNA transfected MSCs.

### eGFP mRNA Transfection

**Figure 2:**
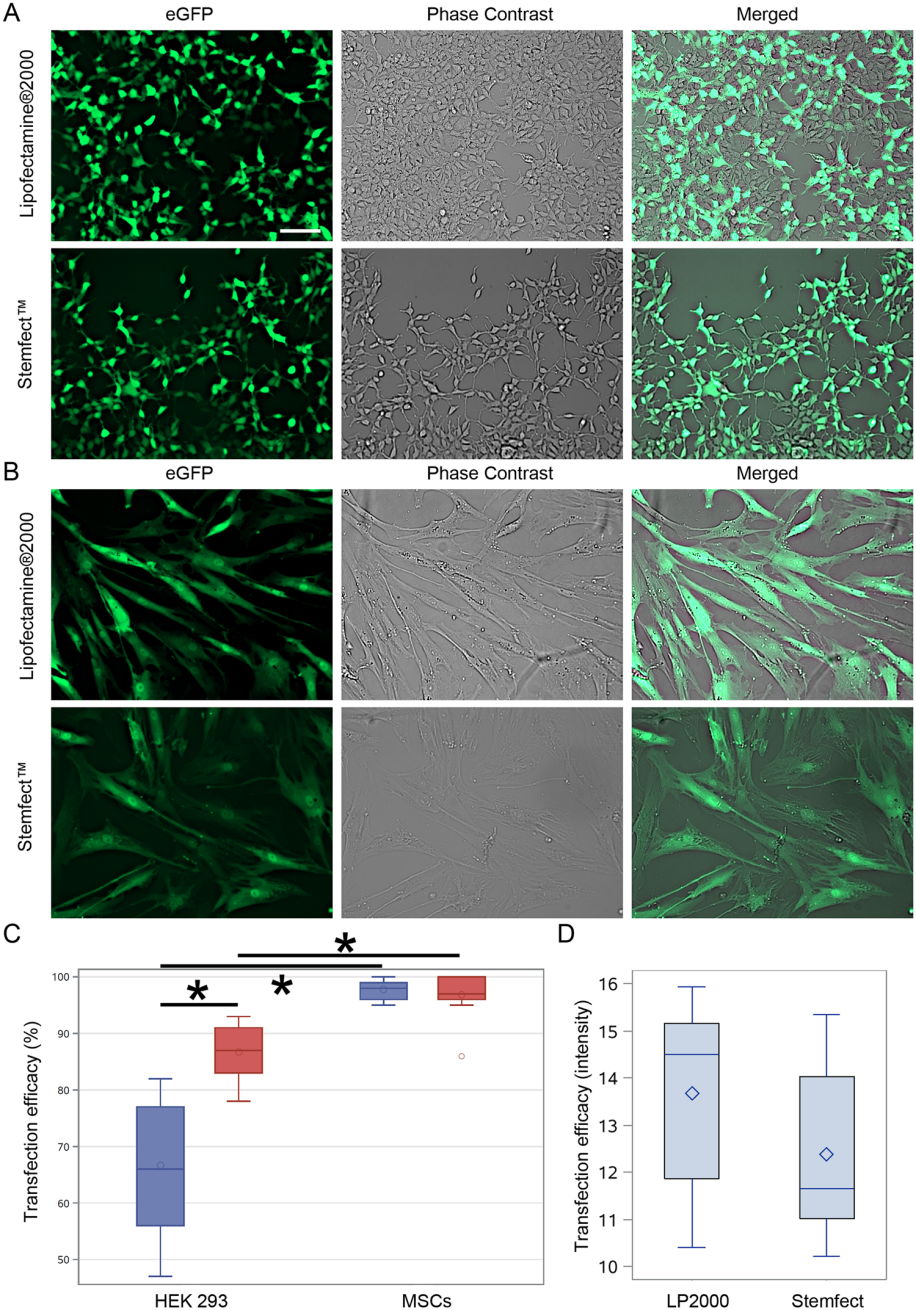
mRNA-based transfections of MSCs and HEK293 cells. The eGFP-mRNA transfection of HEK293 (**A**) and MSCs (**B**) with different transfection agents. Quantification of percentage of cells expressing transgenes (**C**). Quantification of pixel intensity in MSCs transfected with different agents (**D**). Green – eGFP protein. Error bars are box-and-whiskers plots containing the mean, quartiles (box) and minimum and maximum observations (whiskers). Transfection efficiencies have been calculated from three independent experiments. Scale bar located in the top left image: 20 µm.

**Figure 3:**
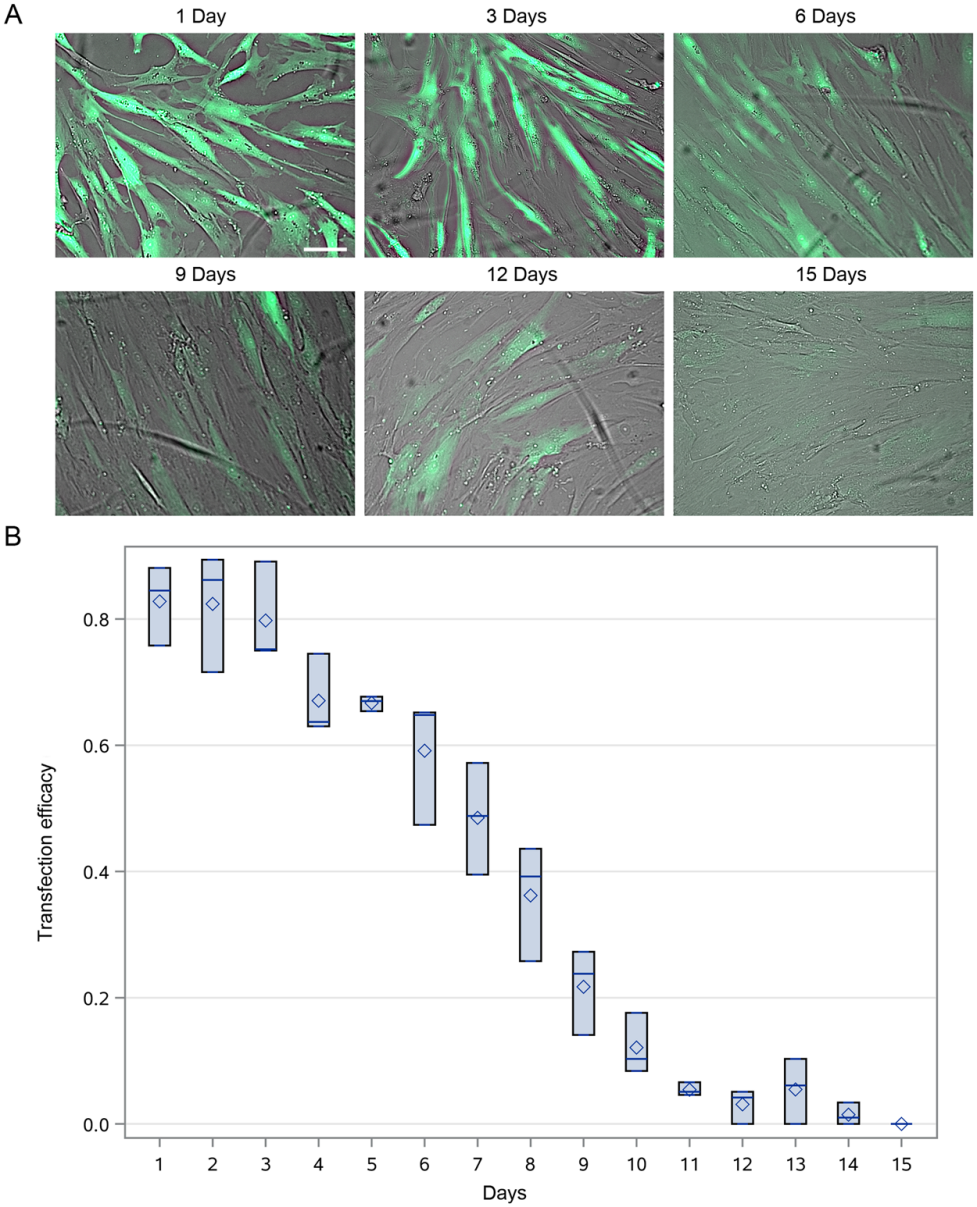
Time-course eGFP protein synthesis after mRNA transfection in MSCs. Time-course of eGFP protein presence in MSCs after eGFP-mRNA transfection on intravital fluorescent images (**A**) with subsequent quantification (**B**). Green – eGFP protein. Error bars are box-and-whiskers plots containing the mean, quartiles (box) and minimum and maximum observations (whiskers). Transfection efficiencies have been calculated from three independent experiments. Scale bar located in the top left image: 20 µm.

In contrast to DNA-based transfections, after eGFP-mRNA (Stemgent) delivery, we observed both eGFP protein positive HEK293 and MSCs. In particular, we found higher number of eGFP protein positive HEK293 cells using Stemfect™ (Stemgent) transfection agent (87%), than Lipofectamine^®^2000 (Invitrogen) (67%) (p < 0.05) (Fig. 2A), while no significant difference was observed in case of MSCs: Stemfect – 97%, and Lipofectamine – 98% (p = NS) (Fig. 2B). Surprisingly, more MSCs were positive for eGFP protein than HEK293 cells, which was observed for both transfection agents (Fig. 2C). No difference in level of eGFP expression in MSCs between both transfection compounds was observed (Fig. 2D). Furthermore, the eGFP expression based on eGFP-mRNA and Lipofectamine^®^2000 was surprisingly long lasting in MSCs and noted up to 10 days (p < 0.05) (Fig. 3).

**Figure 4:**
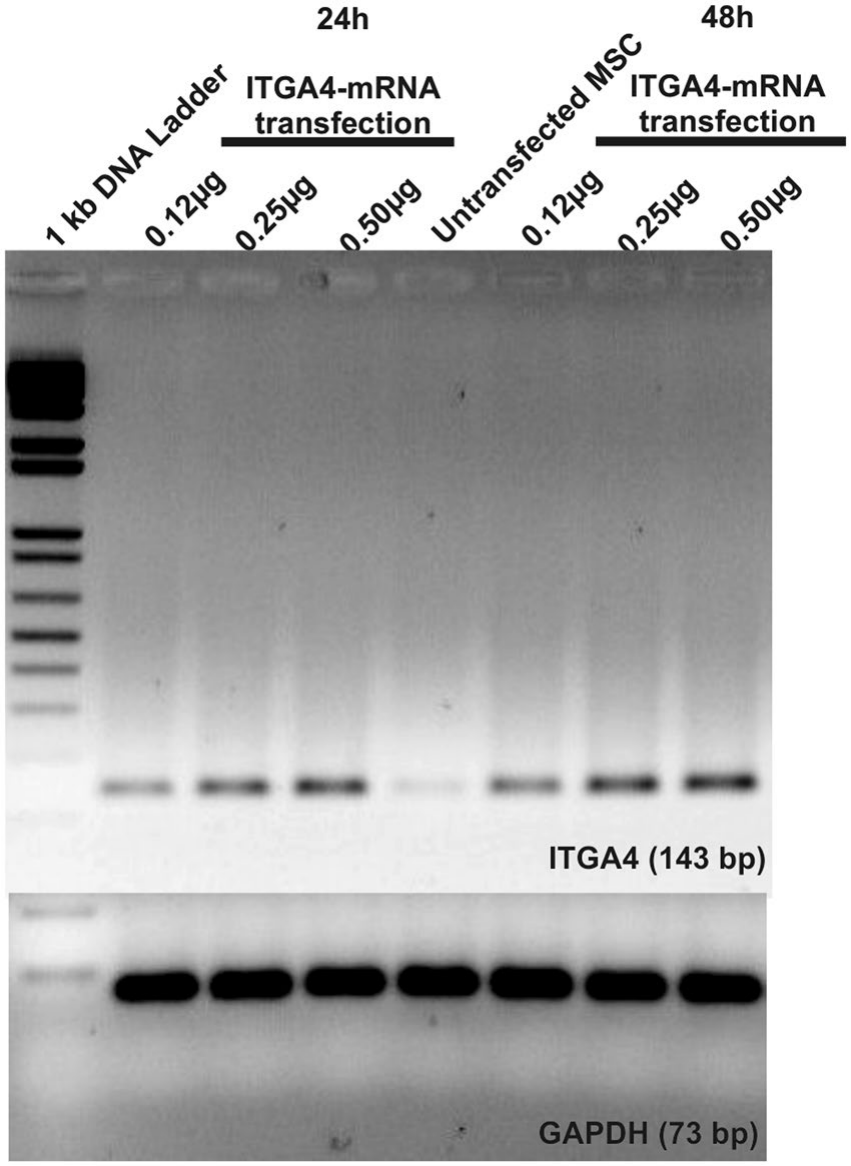
Exogenous ITGA4-mRNA delivery to MSCs. RT-PCR of MSC lysates after ITGA4-mRNA transfection: The higher band refers to ITGA4-mRNA (143 bp) and lower band to housekeeping gene: GAPDH (73 bp).

Based on these results, we designed a plasmid template containing *itga4* cDNA for mRNA production *in vitro* (Suppl. Fig. 2). MSCs and HEK293 cells were transfected with ITGA4-mRNA bearing conventional cap analogue (7-methylguanosine) and 8 h after transfection neither HEK293 (Suppl. Fig. 3A) nor MSCs (Suppl. Fig. 3B) were positive for ITGA4 protein. As in case of DNA plasmid for *itga4*, also mRNA for *itga4* was successfully delivered to the target cells (Fig. 4). This indicates that also not transfection itself, but translation was a major roadblock to achieve efficient production of ITGA4 protein in MSCs and also HEK293 cells.

### Effect of SSB proteins on ITGA4-mRNA translation in MSCs

**Figure 5:**
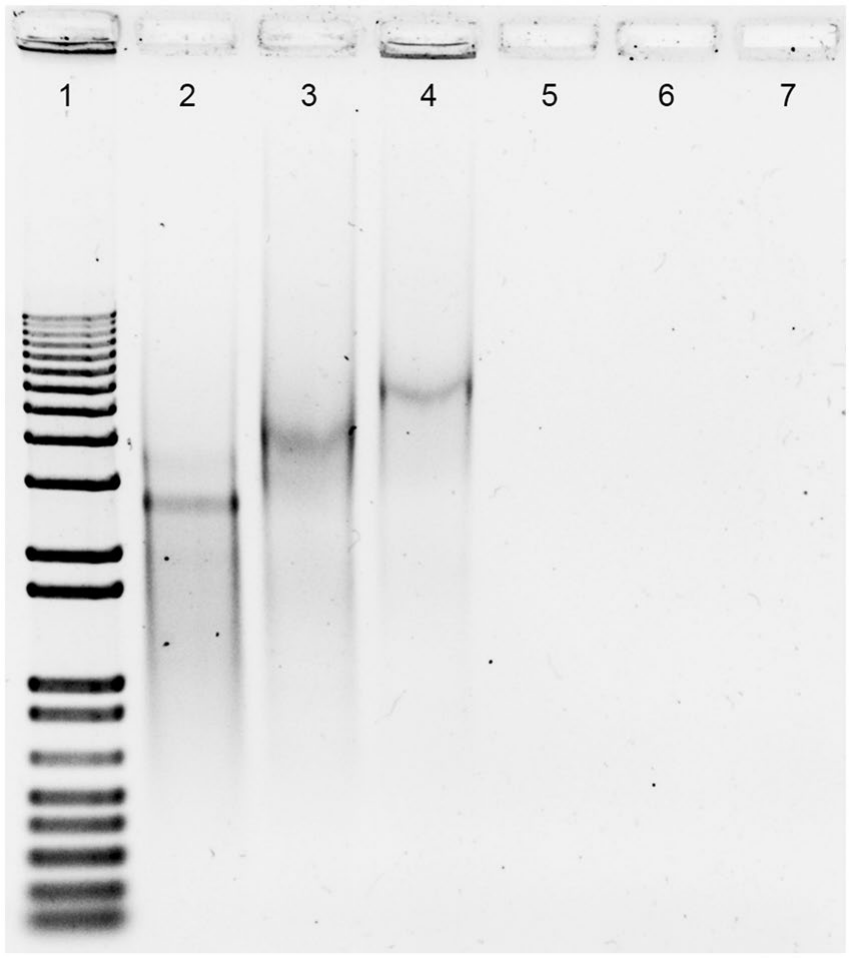
SSB protein binding assay *in vitro*. Electrophoresis after SSB protein to ITGA4-mRNA *in vitro* binding assay: 1) 1 kb DNA ladder, 2) ITGA4-mRNA, 3) ITGA4-mRNA/SSB, 4) ITGA4-mRNA/SSB/Lipofectamine^®^2000, 5) SSB/Lipofectamine^®^2000, 6) SSB, 7) Lipofectamine®2000.

**Figure 6:**
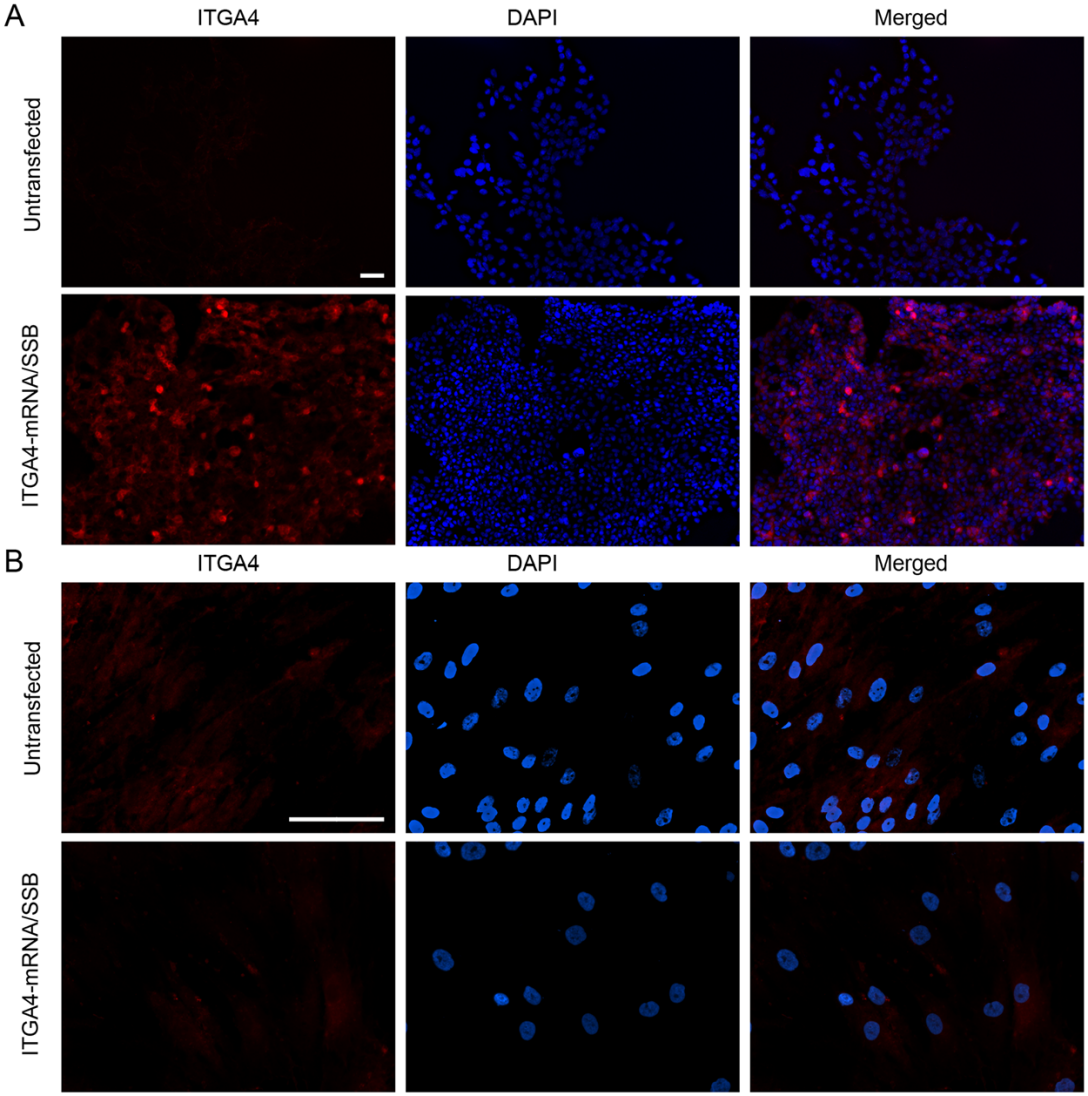
ITGA4 protein synthesis after ITGA4-mRNA/SSB transfection. Immunocytochemistry study for assessment of the ITGA4 protein presence after ITGA4-mRNA/SSB transfection using Lipofectamine^®^2000. Scale bars separate for HEK293 and MSC cells located in the respective left upper images of the panel indicate 100 µm.

In order to achieve effective ITGA4-mRNA translation we investigated the use of single-stranded binding (SSB) nucleic acid proteins. The SSB protein was shown to interact with the ITGA4-mRNA evidenced by change in the electrophoretic migration pattern with retarded migration of the putative ITGA4-mRNA/SSB proteins complexes (Fig. 5). These observations prompted us to conduct MSCs and HEK293 cells transfections with ITGA4-mRNA/SSB protein complexes. ITGA4 protein was detected 5 h after lipofection in HEK293 cells (Fig. 6A), but not in MSCs in any time point up to 72 hours (Fig. 6B).

### Effect of ARCA 5′-cap analogue on ITGA4-mRNA MSCs transfection efficacy

**Figure 7:**
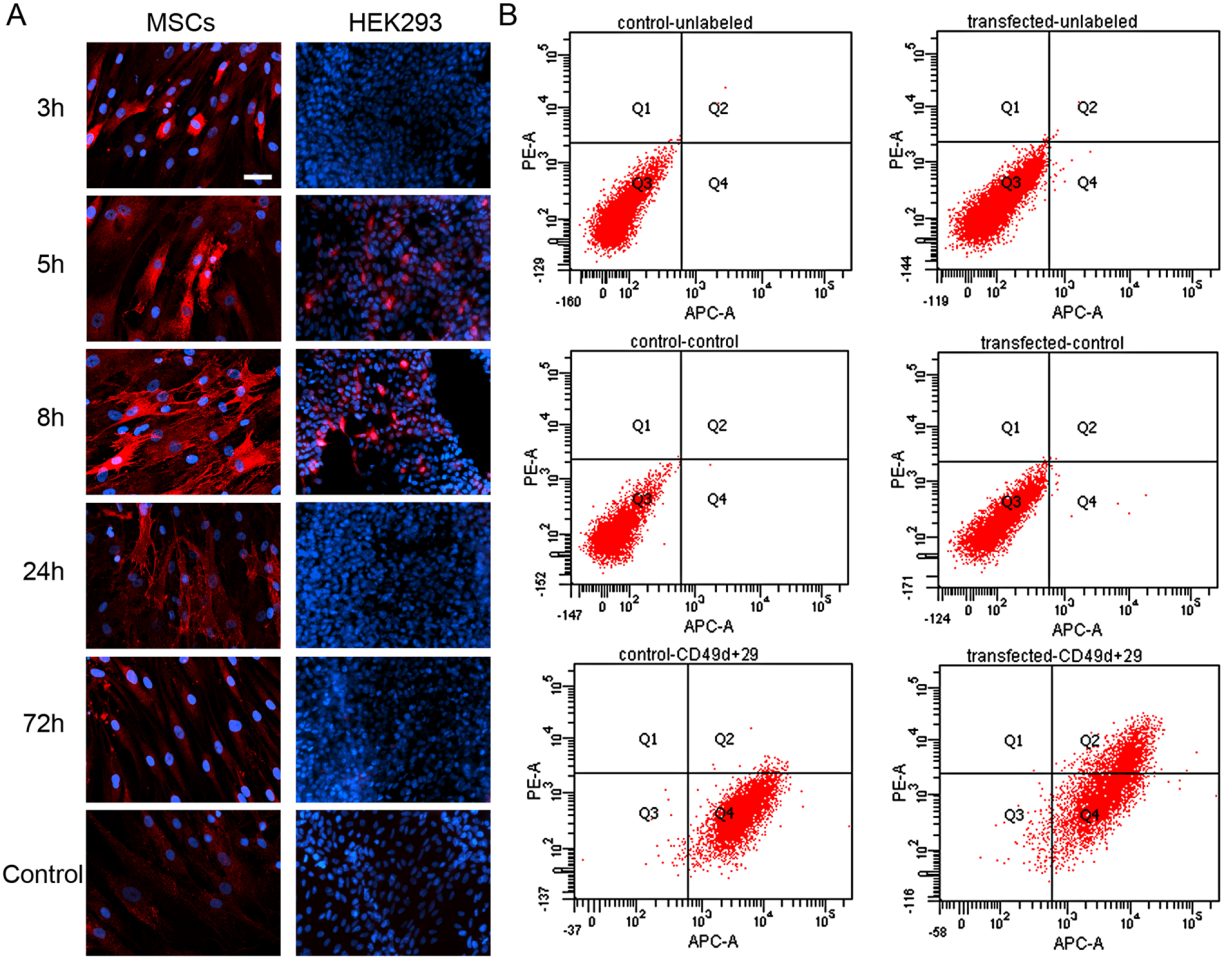
ITGA4 protein distribution in (ARCA)-ITGA4-mRNA transfected MSCs. Time-course of ITGA4 protein production – note its translocation from perinuclear area in 3 and 5 hour time-points toward the cell membrane in 8 and 24 hour time points (**A**). The presence of ITGA4 protein on the cell membrane was confirmed in living cells by flow cytometry performed 20 h after transfection (**B**). red – ITGA4 protein, blue – Hoechst 33258. Scale bar: 20 µm.

**Figure 8:**
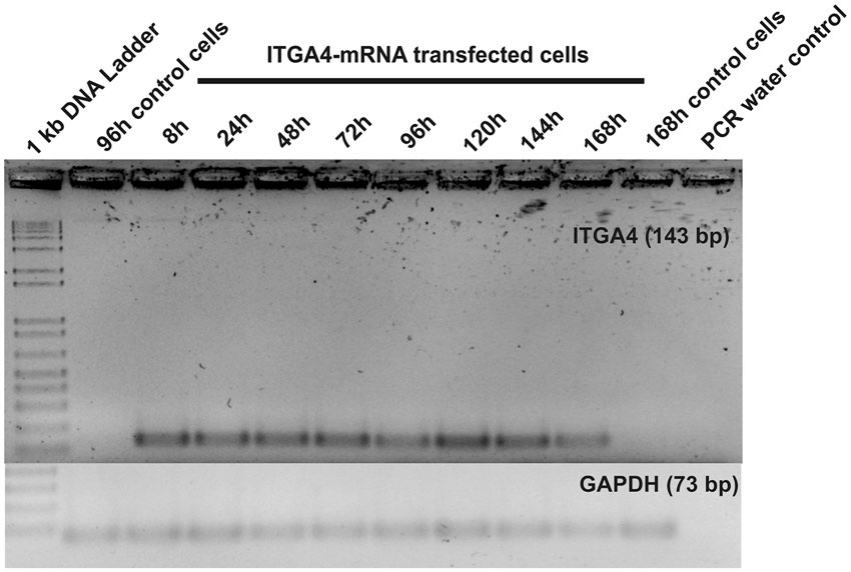
Longitudinal study of ITGA4-mRNA presence in transfected MSCs. RT-PCR results from MSCs transfected with (ARCA)-ITGA4-mRNA confirmed that delivered (ARCA)-ITGA4-mRNA was present in transfected cells at least for 168 h.

To further investigate the effective method for ITGA4-mRNA translation we employed an anti-reverse cap analogue (ARCA) instead of conventional cap analogue (7-methylguanosine). (ARCA)-ITGA4-mRNA was efficiently produced *in vitro*, and HEK293 cells as well as MSCs were transfected subsequently. The ITGA4 protein was detected in both cell types. The protein distribution pattern in MSCs showed gradual transition from intracellular compartments (3 and 5 h) towards the outer cell membrane (8 and 24 h) (Fig. 7A), and transfection efficiency was calculated to be around 75,1 +/− SD 14,6% (construed as whole ITGA4 positive MSCs regardless of the ITGA4 protein distribution in the cellular compartments 8 h after transfection by the immunocytochemical evaluation). The effective docking of ITGA4 to the membrane has been confirmed by flow cytometry using anti-ITGA4 antibody in living cells (Fig. 7B). After reaching the cell membrane, the cytoplasmic presence of ITGA-4 phased out with the signal present only in the cell membrane but not in the cytoplasm. The ITGA4 signal disappeared completely up to 72 hours after transfection. Detection of exogenous (ARCA)-ITGA4-mRNA in transfected MSCs confirmed cellular presence of this construct for up to seven days after transfection (Fig. 8) even though protein production lasted only up to 24 hours. It also confirms that translation might be a factor limiting efficiency of mRNA-based production of proteins.

## Discussion

Here, we show expression of ITGA4 protein in MSCs after mRNA-based transfection. Our initial lack of success with plasmid DNA-based methods is in line with experience of others^31, 37^. Transfection efficiency inversely correlates with plasmid size^38^. However, with the experiment using small size 4.7 kb p-eGFP-N1 vector we limited this as a cause for lack of transgene expression. Our subsequent investigations strongly suggested that the problem was not in transfection, taken as a delivery of genetic material into cells, but rather transcription of plasmid DNA into mRNA (Fig. 1). Transcription was particularly inefficient in MSCs. In the process of transfection, DNA is internalized and is initially degraded in lysosomes. Escaping these clearing mechanisms is required for sufficient cytoplasm concentration, nucleus entry and subsequent transcription^39^. We speculate that the process of lysosomal escape is ineffective in MSCs, preventing DNA from further processing. This point, however, requires future investigation.

In light of these unsatisfactory results, mRNA-based transfection was developed. Ryser and colleagues reported that electroporation of CXCR4-mRNA resulted in over 90% positively transfected MSCs^36^. However, electroporation disintegrates cell membrane stability with potential side effects and can reduce cell viability. Thus it is not desired for use in cell populations directly before transplantation. Lipofectamine®2000 was found as an attractive transfection agent for mRNA delivery to MSCs (Figs 2 and 3), ensuring fairly high cell viability^35^ and efficient delivery^40^. However, genes used in these studies were smaller compared to *itga4*, which might explain why the approach was not effective in our hands although successful delivery of nucleic acid to the cells could be shown. Our next strategy was to stabilize exogenous mRNA with the use of SSB proteins (Fig. 5), which failed for MSCs (Fig. 6). Similar SSB protein utility has been described for other more naïve cell populations including tumor cells^41^. To exclude technical problems, we reproduced the result of Shi *et al*. in HEK293 immortalized cells. This result indicates profound differences in mRNA handling between tumor/immortalized cell lines and populations of primary stem cells such as MSCs, which must be taken into account.

The ITGA4 protein synthesis in transfected MSCs was finally achieved by using ARCA-modified ITGA4-mRNA ((ARCA)-ITGA4-mRNA) (Fig. 7). During *in vitro* production of mRNA the conventional cap analogue is incorporated in correct orientation only in 50% of cases. The design of an ARCA cap analogue guarantees incorporation of nucleotides exclusively in the normal/physiological orientation during *in vitro* synthesis. In consequence, it enables recognition by translation initiating proteins. This was an important innovation for extracellular *in vitro* production of functional mRNA^42^. It was found later that ARCA also increases translational efficiency and decays resistance^43^. The abovementioned properties may have contributed to the production of ITGA4 protein in MSCs, particularly through a delay of the 5′-decapping process of (ARCA)-ITGA4-mRNA preventing the mRNA chain from the exposure to exonuclease- mediated degradation^44, 45^.

Although we observed translocation of ITGA4 protein from perinuclear cytoplasm to the membrane (Fig. 7), the expression lasted for a relatively short period of time 24 h, which was much shorter than eGFP expression (up to 10 days). Since mRNA was active directly after transfection, the complete failure of release from lysosomes is relatively unlikely as is a potential “secondary” or delayed entrapment of mRNA in lysosomes. We hypothesize that eGFP is not a native protein in vertebrates, thus human MSCs may be lacking mechanisms for specific silencing of this mRNA. The microRNA may be postulated as such mechanism^46^. In both cases mRNA containing specific sequence may be present in cells for some time, which is in line with our observation that transfected mRNA is present in cells longer than translated to proteins (Fig. 8).

Not only integrins but also selectins participate in the process of diapedesis. It was recently shown that triple gene engineered (including two selectin ligands), intravenously injected MSCs home more efficiently to the spinal cord of animal models what directly translates into therapeutic activity^47^. However, the authors were not able to detect transplanted cells through reporter gene assays and or the used membranous label. The co-localization of this marker with immune cells may indicate that MSC and/or any released label were phagocytosed. Moreover, the authors did not perform vessels staining such as claudin-5 so no information about diapedesis can be drawn. It was previously shown that intravenously injected cells should rather have blocked homing ligands to pass pulmonary capillaries without being filtered^48^. Additionally, MSCs entrapped in lungs release TSG6, what may decrease local inflammation^49^, what may explain therapeutic efficacy without cell detection *in situ* in this particular study. Three mRNAs can be more cumbersome to apply, and less attractive from clinical perspective than using only one mRNA, as in our case.

While long-lasting expression of a transgene may be sought in many applications, in our case the feature of short-term expression may exactly fit requirements. Diapedesis is a fast process^50^, while extended expression of the homing receptors in turn might cause safety concerns rather than providing enhanced therapeutic efficacy. In a hypothetical clinical scenario, the provided MSCs (just thawed or maintained in culture *in vitro*) can be transfected overnight, with most of the cells effectively engineered (no need for sorting as in case of DNA) and transplanted to the patient without major delays. Thus, mRNA-based engineering is a very convenient tool for inducing short-lasting manipulations in therapeutic cells.

## Concluding Remarks

In this study, we demonstrated that plasmid DNA-based transgene delivery to MSCs was ineffective, prompting the use of mRNA-based transfection approaches. However, mRNA based transfection strategy is related to specific problems such as exogenous mRNA stability and recognition by protein production machinery that need to be solved in the first instance. We further demonstrated here that not transfection but rather translation efficiency of exogenous mRNA seems to be the major obstacle in efficient mRNA-based engineering of MSCs. Methods that are efficient in inducing exogenous expression using mRNA-based transfection in tumor/immortalized cell lines do not necessarily apply to primary stem cells such as MSCs. The use of ARCA 5′-cap analogue resulted with successful production of ITGA4 protein in MSCs. The possibility for overnight mRNA-based ubiquitous engineering of stem cells prior to transplantation is compelling.

## Materials and Methods

### Cell culture

Human mesenchymal stem cells (hMSCs, PT-2501, Lonza) were obtained from randomly selected healthy donors of both sexes with ages ranging from 19 to 38 years old and cultured in dedicated medium MSCBM (PT-3238) supplemented with 10% MCGS (PT-4106E), L-glutamine (PT-4107E), and gentamicin sulfate (GA-1000, PT-4504E, all Lonza), which was changed twice a week. Cells were maintained for up to 9 passages in 75 cm^2^ plastic bottom flasks and split once a week. For transgene induction experiments, cells were transferred to 24-well plates and seeded at a density of 1.5 × 10^4^ cells/well. HEK293 cells served as controls and were cultivated in DMEM medium (31966 GlutaMAX, GIBCO) with 10% FBS (P40-37500HI, PAN™Biotech) and penicillin/streptomycin (P11-010, PAA). Cells were grown in 25 cm^2^ flasks, but for transfections assays were transferred to 24-well plates at a density of 70 × 10^3^ cells/well. Both cell types were cultured in a humidified atmosphere at 37 °C and 5% CO_2_.

### Genetic material

#### Plasmid vectors

A p-eGFP-N1 vector containing the enhanced green fluorescence protein (eGFP) gene was purchased from BD Biosciences (PT 3027-5, BD). A commercially designed vector for mRNA synthesis *in vitro* (2.4 kbp pSP72 plasmid) was obtained from Promega (P2191, Promega). pITGA4 and pITGA4(IRES)eGFP plasmids were produced in our lab (The Johns Hopkins University School of Medicine, Baltimore, by Dr. Walczak).

#### mRNA synthesis *in vitro*

In order to produce mRNA *in vitro*, a fragment containing *itga4* gene cDNA was released from the pITGA4 vector by simultaneous XbaI (FD0684, ThermoScientific) and XhoI (FD0694, ThermoScientific) digestion before being cloned into the pSP72 vector with T4 DNA ligase (EL0014, ThermoScientific). This newly created pITGA4-SP72 vector was sequenced (ABI3730 Genetic Analyzer, Applied Biosystems) and used as a template for the mRNA production *in vitro*. The mMessage mMachine^®^ Kit (AM1344, Ambion^®^), mMessage mMachine^®^ T7 Ultra Kit (AM1345, Ambion^®^) and poly (A) tailing kit (AM1350, Ambion^®^) were used according to the manufacturers’ instructions. Synthesized mRNA was precipitated by LiCl and washed with 70% EtOH, and mRNA concentration was evaluated spectroscopically (NanoDrop^®^ND-1000, ThermoScientific). Control eGFP-mRNA (100 ng/µl) was purchased (05-0020, Stemgent).

#### Single-Strand Binding Protein assay *in vitro*

Single-Strand Binding Protein (SSB) was delivered from Sigma (S3917, Sigma). For *in vitro* RNA/SSB complex formation assays, 0.25 µg of ITGA4-mRNA was incubated with 10 µg of SSB protein with or without 1 µl of Lipofectamine^®^ 2000 (11668-019, Life Technologies) in Opti-Mem^®^ I (1x) Reduced Serum Medium (31985-047, Life Technologies; final volume: 12 µl) for 20 minutes at RT. Then, 1% agarose gel electrophoresis was performed in non-denaturing conditions. 1 kb Plus DNA Ladder (10787-018, Invitrogen) was used as a DNA size marker.

#### Preparation of transfection complex and cells transfections with Lipofectamine® 2000

24 h prior to transfection, MSCs and HEK293 cells were plated on coverslips previously treated with poly-L-lysine 40 µg/ml (P2636, Sigma). Growing medium was removed 30 minutes before transfection, cells were washed with PBS, and 200 µl of Opti-Mem^®^ I (1x) Reduced Serum Medium (31985-047, Life Technologies) was poured per well. 48 µl of Opti-Mem^®^ I (1x) Reduced Serum Medium were prepared in a 1.5 ml sterile plastic tube and 2 µl of Lipofectamine^®^ 2000 (Invitrogen) were added, followed by 5 minutes incubation at room temperature (RT). Meanwhile, 0.12–0.5 µg mRNA or 1.0 µg plasmid DNA was diluted with 50 µl of Opti-Mem^®^ I (1x) Reduced Serum Medium. Controlled cells were processed with the same protocol, but in mRNA-free manner. In variants where SSB protein was used for mRNA stabilization, 0.25 µg of ITGA4-mRNA and 10 µg of SSB protein were applied for the lipoplex formation. Liposome reagent and genetic material samples were mixed for lipoplex formation and incubated for 20 minutes at RT. In the next step, 100 µl of the reaction mix was administrated to the cells followed by 4 h incubation at 37 °C/5% CO_2_. Lipoplex containing medium was removed afterwards and standard growing medium was added.

#### Stemfect™RNA Transfection Kit

Stemfect™RNA Transfection Kit was purchased from Stemgent (00-0069, Stemgent). For transfection, mRNA was diluted with Stemfect Transfection Buffer in total volume of 11.5 µl in a 1.5 ml sterile tube. In a separate tube, 2 µl of Stemfect RNA Transfection Reagent were added to 12.5 µl of Stemfect Transfection Buffer. The contents of these two tubes were mixed and incubated for 10 minutes at RT, followed by drop-let administration to the cells residing in 500 µl of standard growing medium. That growing medium was changed 24 h post transfection.

#### PCR and RT-PCR

For PCR and RT-PCR analysis respectively, cells were collected at 8, 24, 48, 72, 96, 120, 144 and 168 h after transfection, washed with PBS and lysed with TRIzol^®^ Reagent (Ambion^®^). RNA and plasmid DNA samples were obtained by chloroform extraction, followed by precipitation with isopropanol and purification with 75% EtOH. Samples were air-dried and dissolved in RNase-free water. RNA samples were DNase-treated (DNA-free™ Kit AM1906, Ambion^®^) and RNA concentration was measured using the NanoDrop technology (NanoDrop^®^ND-1000, ThermoScientific). High-Capacity RNA-to-cDNA™ Kit (4387406, Applied Biosystems^®^) was used according to the manufacturer’s instructions for Reverse Transcription reactions. For PCR amplifications Taq DNA Polymerase (201205, Qiagen) was employed with the following pairs of primers.

ITGA4-forward: 5′-ACAGATGCAGGATCGGAAAG-3′

ITGA4-reverse: 5′-CCTGGCTGTCTGGAAAGTGT-3′,

GAPDH-forward: 5′-CCACATCGCTCAGACACCAT-3′,

GAPDH-reverse: 5′-TGACCAGGCGCCCAATA-3′.

PCRs were carried out in total volume of 10 µl as follows: initial denaturation for 3 min at 97 °C, followed by 25 cycles (30 sec at 97 °C, 30 sec at 60.3 °C, 45 sec at 72 °C), and final elongation step for 10 min at 72 °C. Subsequent electrophoresis was performed in 2% agarose gels (basica le cgt, Prona) in TAE1x buffer and photos were taken by G:box (Syngene).

#### Immunocytochemistry assays

For immunocytochemistry assays, transfected and control cells were incubated for 3, 5, 8, 24 and 72 h after transfection in a humidified atmosphere at 37 °C and 5% CO_2_. Cells were then fixed with PFA 4% (Roth)/PBS for 10 minutes, followed by permeabilization with 0.3% TritonX (Triton^®^ X-100 Sigma Ultra T9284, Sigma)/PBS for 5 minutes. Samples were blocked with 5% BSA (A9647, Sigma)/PBS and incubated with primary antibody (mouse-monoclonal-IgG_1_ anti-ITGA4 (MAB1354, RD) diluted 1:200 in 0.2% BSA/PBS) solution overnight at 4 °C. After washing with PBS, cells were incubated with goat-anti-mouse IgG(H + L)-AlexaFluor488 (A11001, Invitrogen) for 1 h. Hoechst 33258 (B-2883, Sigma) or DAPI were used as a counterstains. Samples were examined using a Zeiss AX10 microscope and micrographs were analyzed by ZEN (blue edition) software (Carl Zeiss MicroImaging GmbH). In quantitative examinations, at least 1000 cells were counted per each individual experiment or ten randomly selected microscopic fields were selected at x20 or x40 magnification and expressed as a percent of fluorescent cells among all of cells. In the particular case of pITGA4(IRES)eGFP transfection, only ITGA4-positive cells were classified as positive. If no difference in the percent of positive cells was found, the level of gene expression was investigated additionally in order to select the best transfection factor for further experiments. This was done by the measurement of pixel intensity per image with at least 8 images per group were used for quantification.

#### Flow cytometry analysis

Prior to the respective experiment, control and transfected MSCs were cultivated in 25 cm^2^ flasks. Cells were detached by trypsinization, and resuspended in 2 mM EDTA/0.3% BSA/PBS solution. In the next step, cells were labeled using the following antibodies: APC Mouse IgG_1_, κ Isotype Control (555751, BD Pharmingen™), PE Mouse IgG_1_, κ Isotype Control (555749, BD Pharmingen™), APC Mouse Anti-Human CD29 (559883, BD Pharmingen™), PE Mouse Anti-Human CD49d (555503, BD Pharmingen™), washed and analyzed by BD FACSCanto II (BD Bioscences).

#### Statistics

Restricted maximum likelihood approach (PROC MIXED, SAS 9.4) has been used to determine statistical significance which was considered at p < 0.05.

## Electronic supplementary material

Supplementary figures

## References

[1] Janowski M, Wagner DC, Boltze J (2015). Stem Cell-Based Tissue Replacement After Stroke: Factual Necessity or Notorious Fiction?. Stroke..

[2] Kabadi SV, Faden AI (2014). Neuroprotective strategies for traumatic brain injury: improving clinical translation. Int J Mol Sci..

[3] Savitz SI, Cramer SC, Wechsler L (2014). STEPS 3 Consortium. Stem cells as an emerging paradigm in stroke 3: enhancing the development of clinical trials. Stroke..

[4] Nowakowski A, Walczak P, Janowski M, Lukomska B (2015). Genetic Engineering of Mesenchymal Stem Cells for Regenerative Medicine. Stem Cells Dev..

[5] Huang B, Tabata Y, Gao JQ (2012). Mesenchymal stem cells as therapeutic agents and potential targeted gene delivery vehicle for brain diseases. J Control Release..

[6] Domínguez-Bendala J, Lanzoni G, Inverardi L, Ricordi C (2012). Concise review: mesenchymal stem cells for diabetes. Stem Cells Transl Med..

[7] Miura Y (2016). Human bone marrow mesenchymal stromal/stem cells: current clinical applications and potential for hematology. Int J Hematol..

[8] Kim G (2015). Therapeutic Effects of Mesenchymal Stem Cells for Patients with Chronic Liver Diseases: Systematic Review and Meta-analysis. J Korean Med Sci..

[9] Narita T, Suzuki K (2015). Bone marrow-derived mesenchymal stem cells for the treatment of heart failure. Heart Fail Rev..

[10] Lee JS (2010). A long-term follow-up study of intravenous autologous mesenchymal stem cell transplantation in patients with ischemic stroke. Stem Cells..

[11] Bonab MM (2012). Autologous mesenchymal stem cell therapy in progressive multiple sclerosis: an open label study. Curr Stem Cell Res Ther..

[12] Kupcova-Skalnikova H (2013). Proteomic techniques for characterisation of mesenchymal stem cell secretome. Biochimie..

[13] Heo JS (2013). Neural transdifferentiation of human bone marrow mesenchymal stem cells on hydrophobic polymer-modified surface and therapeutic effects in an animal model of ischemic stroke. Neuroscience..

[14] Kim ES (2012). Human umbilical cord blood-derived mesenchymal stem cell transplantation attenuates severe brain injury by permanent middle cerebral artery occlusion in newborn rats. Pediatr Res..

[15] Gutiérrez-Fernández M (2013). Effects of intravenous administration of allogenic bone marrow- and adipose tissue-derived mesenchymal stem cells on functional recovery and brain repair markers in experimental ischemic stroke. Stem Cell Res Ther..

[16] Walczak P (2008). Dual-modality monitoring of targeted intraarterial delivery of mesenchymal stem cells after transient ischemia. Stroke..

[17] Mitkari B (2013). Intra-arterial infusion of human bone-marrow derived mesenchymal stem cells results in transient localization in the brain after cerebral ischemia in rats. Exp Neurol..

[18] Yavagal DR (2014). Efficacy and dose-dependent safety of intra-arterial delivery of mesenchymal stem cells in a rodent stroke model. PLoS One..

[19] Harting MT (2009). Intravenous mesenchymal stem cell therapy for traumatic brain injury. J Neurosurg..

[20] Ho JH (2012). Multiple intravenous transplantations of mesenchymal stem cells effectively restore long-term blood glucose homeostasis by hepatic engraftment and β-cell differentiation in streptozocin-induced diabetic mice. Cell Transplant..

[21] Mohr A (2010). Targeting of XIAP combined with systemic mesenchymal stem cell-mediated delivery of sTRAIL ligand inhibits metastatic growth of pancreatic carcinoma cells. Stem Cells..

[22] Janowski M (2013). Cell size and velocity of injection are major determinants of the safety of intracarotid stem cell transplantation. J Cereb Blood Flow Metab..

[23] Cui L (2015). The cerebral embolism evoked by intra-arterial delivery of allogeneic bone marrow mesenchymal stem cells in rats is related to cell dose and infusion velocity. Stem Cell Research & Therapy..

[24] Vu Q, Xie K, Eckert M, Zhao W, Cramer SC (2014). Meta-analysis of preclinical studies of mesenchymal stromal cells for ischemic stroke. Neurology..

[25] Ley K, Laudanna C, Cybulsky MI, Nourshargh S (2007). Getting to the site of inflammation: the leukocyte adhesion cascade updated. Nat Rev Immunol..

[26] Gorelik M (2012). Use of MR cell tracking to evaluate targeting of glial precursor cells to inflammatory tissue by exploiting the very late antigen-4 docking receptor. Radiology..

[27] Sato H (2005). Epidermal growth factor receptor-transfected bone marrow stromal cells exhibit enhanced migratory response and therapeutic potential against murine brain tumors. Cancer Gene Ther..

[28] Nowakowski A, Andrzejewska A, Janowski M, Walczak P, Lukomska B (2013). Genetic engineering of stem cells for enhanced therapy. Acta Neurobiol Exp (Wars)..

[29] Cho JW, Lee CY, Ko Y (2012). Therapeutic potential of mesenchymal stem cells overexpressing human forkhead box A2 gene in the regeneration of damaged liver tissues. J Gastroenterol Hepatol..

[30] Elsler S (2012). Effective, safe, nonviral gene transfer to preserve the chondrogenic differentiation potential of human mesenchymal stem cells. J Gene Med..

[31] Madeira C (2010). Nonviral gene delivery to mesenchymal stem cells using cationic liposomes for gene and cell therapy. J Biomed Biotechnol..

[32] Park SJ, Na K (2012). The transfection efficiency of photosensitizer-induced gene delivery to human MSCs and internalization rates of eGFP and Runx2 genes. Biomaterials..

[33] Majumdar MK (2003). Characterization and functionality of cell surface molecules on human mesenchymal stem cells. J Biomed Sci..

[34] Kumar S, Ponnazhagan S (2007). Bone homing of mesenchymal stem cells by ectopic alpha 4 integrin expression. FASEB J..

[35] Rejman J (2010). mRNA transfection of cervical carcinoma and mesenchymal stem cells mediated by cationic carriers. J Control Release..

[36] Ryser MF (2008). mRNA transfection of CXCR4-GFP fusion—simply generated by PCR-results in efficient migration of primary human mesenchymal stem cells. Tissue Eng Part C Methods..

[37] Escoffre JM, Teissié J, Rols MP (2010). Gene transfer: how can the biological barriers be overcome?. J Membr Biol..

[38] Ribeiro S (2012). Plasmid DNA size does affect nonviral gene delivery efficiency in stem cells. Cell Reprogram..

[39] Lechardeur D, Verkman AS, Lukacs GL (2005). Intracellular routing of plasmid DNA during non-viral gene transfer. Adv Drug Deliv Rev..

[40] Levy O (2013). mRNA-engineered mesenchymal stem cells for targeted delivery of interleukin-10 to sites of inflammation. Blood..

[41] Shi H (2013). Systematic functional comparative analysis of four single-stranded DNA-binding proteins and their affection on viral RNA metabolism. PLoS One..

[42] Stepinski J, Waddell C, Stolarski R, Darzynkiewicz E, Rhoads RE (2001). Synthesis and properties of mRNAs containing the novel “anti-reverse” cap analogs 7-methyl(3′-O-methyl)GpppG and 7-methyl (3′-deoxy)GpppG. RNA..

[43] Grudzien-Nogalska E (2007). Synthesis of anti-reverse cap analogs (ARCAs) and their applications in mRNA translation and stability. Methods Enzymol..

[44] Simon E, Camier S, Seraphin B (2006). New insights into the control of mRNA decapping. Trends Biochem Sci..

[45] Nagarajan VK, Jones CI, Newbury SF, Green PJ (2013). XRN 5′–3′ exoribonucleases: structure, mechanisms and functions. Biochim Biophys Acta..

[46] Nelson P, Kiriakidou M, Sharma A, Maniataki E, Mourelatos Z (2003). The microRNA world: small is mighty. Trends Biochem Sci..

[47] Liao W (2016). Mesenchymal stem cells engineered to express selectin ligands and IL-10 exert enhanced therapeutic efficacy in murine experimental autoimmune encephalomyelitis. Biomaterials..

[48] Wang S (2015). Excess Integrins Cause Lung Entrapment of Mesenchymal Stem Cells. Stem Cells..

[49] Lee RH (2009). Intravenous hMSCs improve myocardial infarction in mice because cells embolized in lung are activated to secrete the anti-inflammatory protein TSG-6. Cell Stem Cell..

[50] von Wedel-Parlow M (2011). Neutrophils cross the BBB primarily on transcellular pathways: an *in vitro* study. Brain Res..

