# Supplementary material for: Translation, but not transfection limits clinically relevant, exogenous mRNA based induction of alpha-4 integrin expression on human mesenchymal stem cells
Source: Sci Rep. 2017 Apr 24;7:1103. doi: 10.1038/s41598-017-01304-3 (PMC5430815; doi:10.1038/s41598-017-01304-3)
Supplement: Supplementary file 1 — Supplementary figures [file 41598_2017_1304_MOESM1_ESM.pdf]

Translation, but not transfection limits clinically relevant, exogenous mRNA based induction of alpha-4 integrin expression on human mesenchymal stem cells

Adam Nowakowski[1], Anna Andrzejewska[1], Johannes Boltze[2,3,4,5], Franziska Nitzsche[2,3], Li-li Cui6, Jukka Jolkkonen[6,7], Piotr Walczak[7,8,9], Barbara Lukomska[1], Miroslaw Janowski[1,8,10]

1 NeuroRepair Department, Mossakowski Medical Research Centre, Polish Academy of Sciences, Warsaw, Poland, 2 Fraunhofer-Institute for Cell Therapy and Immunology, Department of Cell Therapy, University of Leipzig, Leipzig, Germany, 3 Translational Centre for Regenerative Medicine, University of Leipzig, Leipzig, Germany, 4 Fraunhofer Research Institution of Marine Biotechnology, Department of Translational Medicine, University of Lubeck, Lubeck, Germany, 5 Cell Technology, Lübeck, German; Institute for Medical and Marine Biotechnology, University of Lübeck, Lübeck, Germany, 6 Stroke Recovery Laboratory, Institute of Clinical Medicine - Neurology, University of Eastern Finland, Kuopio, Finland, 7 Neurocenter, Neurology, University Hospital of Kuopio, Kuopio, Finland, 8 Russell H. Morgan Dept. of Radiology and Radiological Science, Division of MR Research, 9 Institute for Cell Engineering, The Johns Hopkins University School of Medicine, Baltimore, USA, 10 Department of Radiology, Faculty of Medical Sciences, University of Warmia and Mazury, Olsztyn, Poland

**Corresponding author: mjanows1@jhmi.edu*

running head: mRNA-based approach to alpha-4 integrin

keywords: mRNA, ARCA, transfection, stroke, mesenchymal stem cells

Supplementary figure 1. Overview of the experiments (graphical abstract)

Supplementary figure 2. pITGA4-SP72 plasmid template DNA preparation with subsequent verification of ITGA4-mRNA production *in vitro*

E.coli were transfected with pITGA4-SP72 and obtained selected clones were digested with XbaI/XhoI to confirm proper subcloning from pITGA4 to pSP72 (A). The cloning site was verified by sequencing (B). Selected clone pITGA4-SP72 was employed for ITGA4-mRNA production *in vitro* (C).

Supplementary figure 3. ITGA4 protein synthesis after ITGA4-mRNA transfection

Immunocytochemical assessment of ITGA4 protein presence after ITGA4-mRNA bearing natural 7-methylguanosine cap transfection using Lipofectamine®2000.

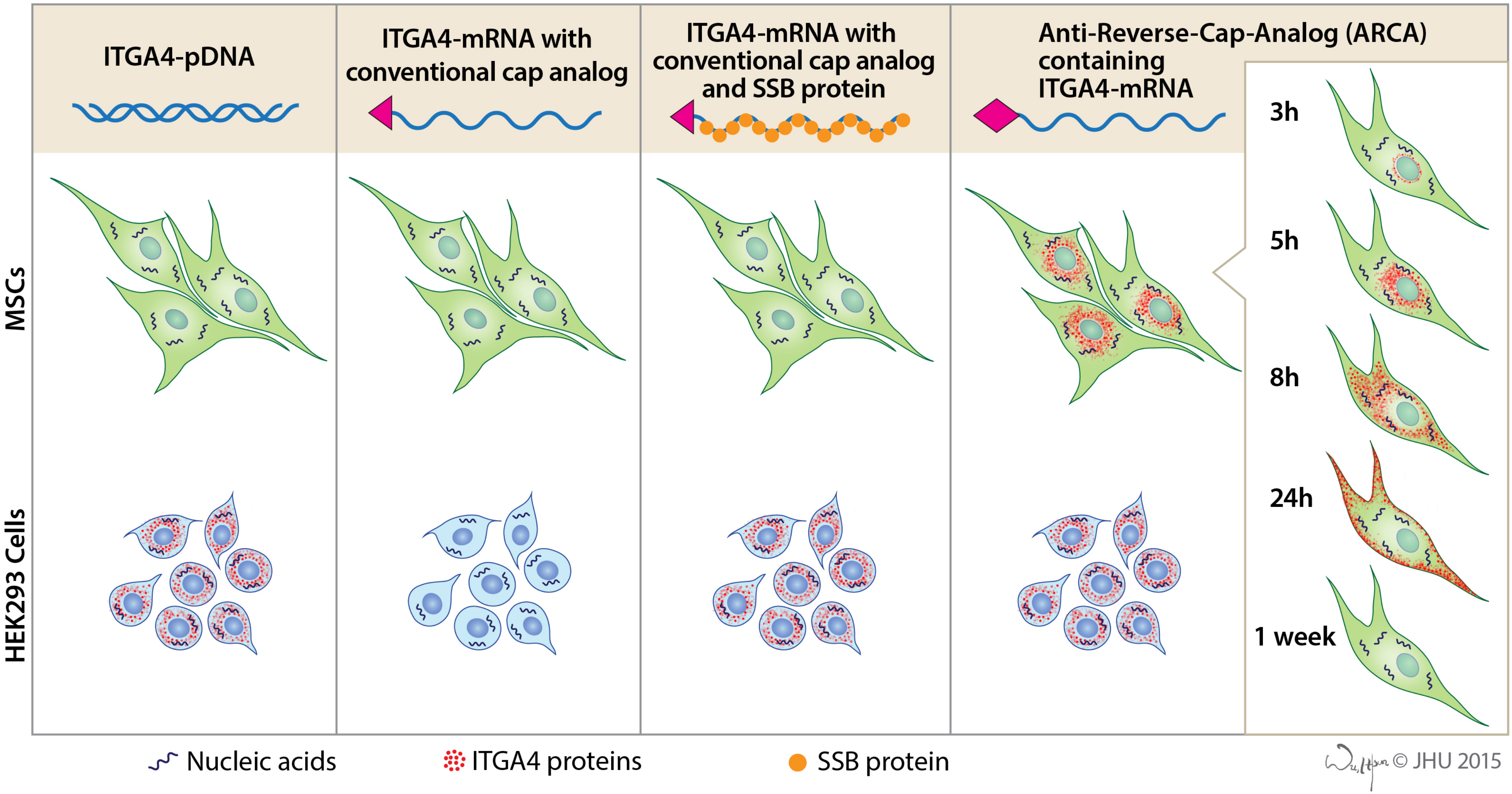
ITGA4-pDNA
ITGA4-mRNA with conventional cap analog
ITGA4-mRNA with conventional cap analog and SSB protein
Anti-Reverse-Cap-Analog (ARCA) containing ITGA4-mRNA
3h
5h
8h
24h
1 week
MSCs
HEK293 Cells
Nucleic acids
ITGA4 proteins
SSB protein
© JHU 2015

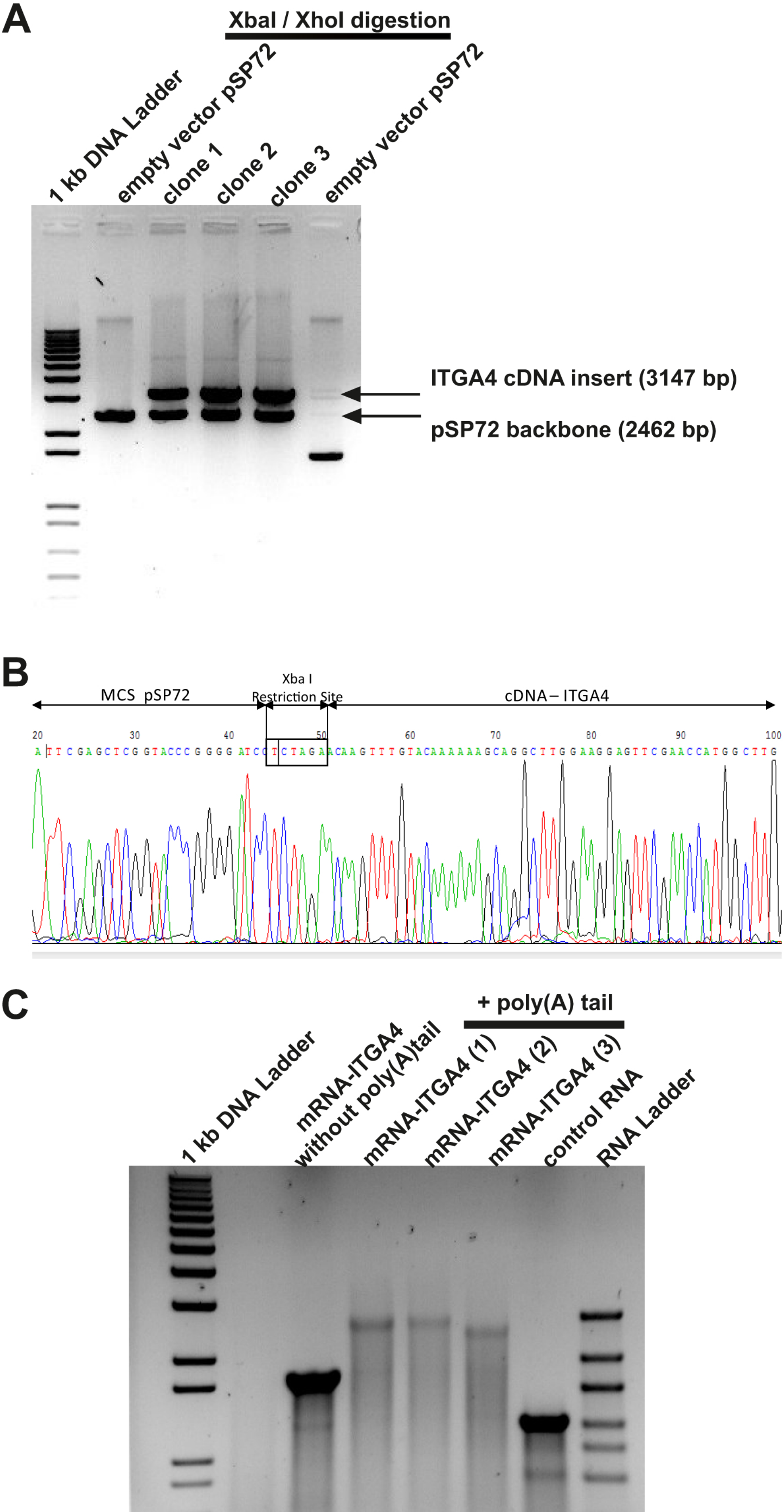
A
XbaI / XhoI digestion
1 kb DNA Ladder
empty vector pSP72
clone 1
clone 2
clone 3
empty vector pSP72
ITGA4 cDNA insert (3147 bp)
pSP72 backbone (2462 bp)
B
Xba I
Restriction Site
MCS pSP72
cDNA – ITGA4
20
30
40
50
60
70
80
90
100
ATTCGAGCTCGGTACCCGGGATCCTCTAGAACAAGTTTGTACAAAAAAGCAGGCTTGGAAGGAGTTCGAACCATGGCTTG
C
+ poly(A) tail
1 kb DNA Ladder
mRNA-ITGA4 without poly(A)tail
mRNA-ITGA4 (1)
mRNA-ITGA4 (2)
mRNA-ITGA4 (3)
control RNA
RNA Ladder

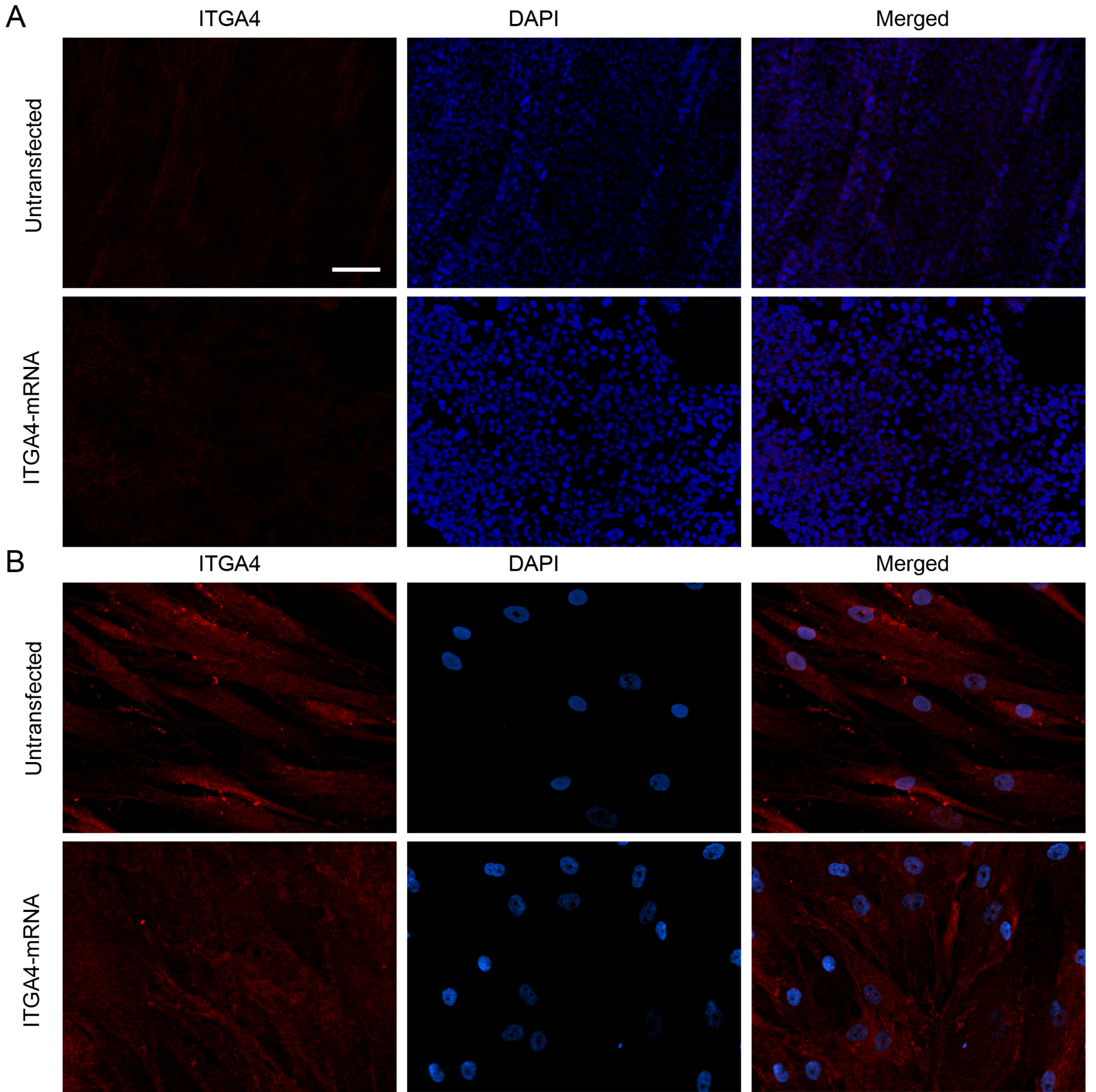
A
ITGA4
DAPI
Merged
Untransfected
ITGA4-mRNA
B
ITGA4
DAPI
Merged
Untransfected
ITGA4-mRNA
